# Association of small intestinal bacterial overgrowth with Parkinson’s disease: a systematic review and meta-analysis

**DOI:** 10.1186/s13099-021-00420-w

**Published:** 2021-04-16

**Authors:** Xiaoqing Li, Xin Feng, Zhongxiang Jiang, Zheng Jiang

**Affiliations:** Department of Gastroenterology, The First Affiliated Hospital of Chongqing Medical University, Chongqing, 400016 China

**Keywords:** Small intestinal bacterial overgrowth, Parkinson’s disease, Systematic review, Meta-analysis

## Abstract

**Objective:**

Parkinson’s disease (PD) is the second most prevalent neurodegenerative disease after Alzheimer's disease (AD) worldwide. The prevalence of small intestinal bacterial overgrowth (SIBO) in PD patients is high. We conducted this comprehensive systematic review and meta-analysis to determine the association between SIBO and PD.

**Methods:**

A comprehensive literature search of the PubMed, Cochrane Library and EMBASE databases was performed to identify studies correlating SIBO with PD. Studies were screened, and relevant data were extracted and analysed. We calculated the pooled prevalence of SIBO in all individuals with PD and compared the prevalence of SIBO between the two groups to calculate an odds ratio (OR) and 95% confidence interval (CI). Egger’s test was performed to assess publication bias.

**Results:**

Eleven studies with 973 participants met the inclusion criteria. The pooled prevalence of SIBO in patients with PD was 46% (95% CI 36–56). A random-effects model was applied given the heterogeneity (I^2^ = 83%) detected among the studies. Egger’s test indicated no publication bias (p = 0.0657). Subgroup analyses showed that the prevalence of SIBO was greater in studies including patients diagnosed using the lactulose hydrogen breath test (LBT) (51%, 95% CI 37–65) than in those including patients diagnosed using the glucose hydrogen breath test (GBT) (35%, 95% CI 20–50), and the prevalence of SIBO in PD was highest (55%, 95% CI 38–72) in patients diagnosed by the LBT and GBT. The prevalence of SIBO was 52% (95% CI 40–64) among patients from Western countries and 33% (95% CI 22–43) among patients from Eastern countries. The pooled OR of SIBO in PD patients compared with healthy controls was 5.22 (95% CI 3.33–8.19, p < 0.00001). We did not identify an obvious predictor of SIBO in PD patients.

**Conclusion:**

In conclusion, our meta-analysis found a strong association between SIBO and PD with approximately half of PD patients testing positive for SIBO. These relationships significantly differed based on diagnostic test and geographic area.

## Introduction

Parkinson’s disease (PD) is the second most prevalent neurodegenerative disease after Alzheimer's disease (AD) worldwide. The neuropathologic changes of PD include abnormal accumulation of alpha-synuclein and degenerative necrosis of dopaminergic neurons in the substantia nigra [1]. The pathogenesis of PD remains unclear; it is generally believed that it may be related to the environment, ageing, heredity and other factors [2]. The symptoms are the classic triad of Parkinsonian motor features: bradykinesia, resting tremor and rigidity. In addition to the above motor-related manifestations, PD patients often have nonmotor symptoms, such as anosmia, sleep disorders, depression and constipation [3]. PD affects the nerves of the entire gastrointestinal (GI) tract, and most PD patients experience abnormal gastrointestinal motility and delayed gastric emptying. Recent research has shown changes in the intestinal microbiota of PD patients, which is associated with the clinical phenotype of PD [4].

Small intestinal bacterial overgrowth (SIBO) is defined as an increased bacterial density greater than 10^5^ colony-forming units/mL and/or abnormal types of bacteria in the small intestinal tract [5]. SIBO can cause nonspecific symptoms, such as bloating, abdominal pain, diarrhoea and weight loss. The gold standard for diagnosing SIBO is a microbial investigation of jejunal aspirate culture (JAC). Noninvasive tests, such as the lactulose hydrogen breath test (LBT) and glucose hydrogen breath test (GBT), are also used for the diagnosis of SIBO [6]. A large number of studies have confirmed that SIBO may be connected to some central nervous system diseases, such as multiple sclerosis, AD and epilepsy. New work suggests a strong association between PD and SIBO. The prevalence of SIBO in PD reported in recent research was 34% [7]. However, current studies do not provide explicit evidence to confirm the correlation at home and abroad. Therefore, we conducted this comprehensive systematic review and meta-analysis to determine the association between PD and SIBO.

## Materials and Methods

### Information sources and search strategy

In this systematic review and meta-analysis, studies on the association of SIBO and PD were searched in PubMed, EMBASE and the Cochrane Library from inception to February 2021. The search terms were as follows: (Parkinson OR Parkinson's disease OR Parkinson's syndrome) AND (small intestinal bacterial overgrowth OR small intestine bacterial overgrowth OR SIBO OR small bowel bacterial overgrowth OR SBBO OR breath test OR lactulose hydrogen OR glucose hydrogen OR jejunal aspirate). References in the articles were assessed to retrieve additional potentially relevant studies. There were no language restrictions.

### Study selection

Studies were included if they met the following criteria: (a) studies that had a cross-sectional, cohort or case–control design; (b) studies that recruited subjects who met the PD diagnostic criteria; (c) studies in which SIBO was diagnosed with the following tests: the GBT, LBT or JAC; (d) studies that compared the association of PD and SIBO; and (e) studies with full texts available. The exclusion criteria were as follows: (a) case reports, review articles, and letters; (b) animal studies; and (c) studies reporting unclear data.

### Data extraction and quality assessment

Two authors (XQ Li and X Feng) independently extracted the following data from each study: first author, year of publication, country, study design, SIBO diagnostic test, prevalence of SIBO in PD patients, SIBO diagnostic criteria, age, sex, course of disease, and quality assessment. The data were reviewed by a third author (Z Jiang). The quality of a cohort study or case control study was evaluated based on the following three domains using the Newcastle–Ottawa scale: the selection of subjects, the comparability of the groups and the determination of the outcome of interest [8]. The quality of a cross-sectional study was assessed using the modified Newcastle–Ottawa scale [9]. Studies with a score ≥ 7 were considered high-quality studies, whereas those with a score < 7 were considered poor quality.

### Statistical analysis

The data were analysed in Review Manager (RevMan) version 5.3. We calculated the pooled prevalence of SIBO in all individuals with PD and compared the prevalence of SIBO between the two groups in cohort and case–control studies to calculate an odds ratio (OR) and 95% confidence interval (CI). Here, p-values less than 0.05 were considered statistically significant. We assessed heterogeneity using the I^2^ statistic. Fixed- and random-effects statistical models were performed for data analysis. If there was high heterogeneity, subgroup analyses were performed to analyse the sources of heterogeneity. Egger’s test was used to assess publication bias, and p > 0.05 in the Egger’s test was considered to indicate no publication bias.

## Results

**Fig. 1:**
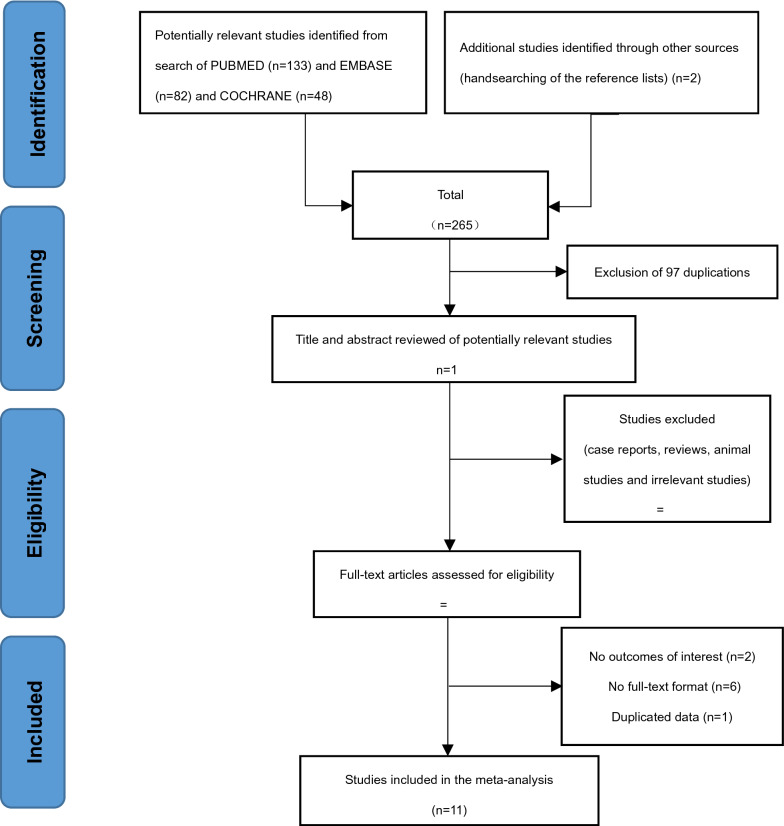
Flow chart of the literature review process

A total of 265 potentially eligible articles were identified based on the described search strategy. Of all the extracted articles, 245 articles were excluded because they were duplicates, reviews, or irrelevant studies. Finally, 11 studies [7, 10–19] (7 cross-sectional studies and 4 cohort studies) with 973 participants (692 PD patients and 281 controls) met the inclusion criteria (Fig. 1). The characteristics and quality evaluation of the included literatures are shown in Table 1. Ten of the 11 articles [7, 10–18] were considered high quality, and one [19] was considered low quality. All the included articles were available in English.

**Table 1:** Main characteristics of the studies included in this meta-analysis

Study	Country	Study design	SIBO diagnostic test	Prevalence of SIBO in Parkinson's patients	SIBO diagnostic criteria	Age (years)	Gender (male/female)	Disease course (years)	Quality assessment
Hasuike et al. [10], 2020	Japan	cross‐sectional	LBT	19/39(48.7%)	10 g lactulose load is orally administered, H2 increase > 20 ppm above basal value within the first 2 h	Overall 66.9 ± 10.4 SIBO + : 64.9 ± 6.3 SIBO-: 68.0 ± 5.4	Overall 17/22 SIBO + : 9/10 SIBO-: 7/9	Overall: 7.2 ± 5.1 SIBO + : 6.5 ± 2.0 SIBO-: 7.6 ± 2.5	Selection: 3 Comparability: 2 Outcome: 2
DiBaise et al. [11], 2018	USA	cross‐sectional	GBT	12/51(23.5%)	not stated	not stated	32/19	not stated	Selection: 3 Comparability: 2 Outcome: 2
Su et al. [7], 2017	USA	cross‐sectional	LBT	22/64(34.4%)	10 g lactulose load is orally administered, H2 increase > 20 ppm on 2 distinct peaks within 1.5 h	72 (52–91)	33/31	6.5 (0.6–22)	Selection: 4 Comparability: 2 Outcome: 2
Niu et al. [12], 2016	China	cohort study	GBT	cases: 55/182(30.2%) controls:19/200(9.5%)	50 g glucose load is orally administered, H2 increase > 20 ppm or CH4 increase > 10 ppm above basal value within the first 1 h	cases: 65 (50–74) SIBO + : 66 (50–75) SIBO-: 64 (50–74) controls: 65 (51–74)	cases: 104/78 SIBO + : 30/25 SIBO-: 74/53 controls: none	cases: 7 (3–12) SIBO + : 10 (5–15) SIBO-: 6 (2–10)	Selection: 4 Comparability: 2 Outcome: 2
Tan et al. [13], 2014	Malaysia	cross‐sectional	LBT	26/103 (25.2%)	10 g lactulose load is orally administered, H2 increase > 10 ppm or CH4 increase > 20 ppm above basal value within the first 1.5 h	Overall 65.4 ± 8.5 SIBO + : 67.2 ± 10.1 SIBO-: 64.8 ± 8.0	Overall 62/41 SIBO + : 16/10 SIBO-: 46/31	Overall 7.3 ± 5.3 SIBO + : 5.2 ± 4.1 SIBO-: 8.1 ± 5.5	Selection: 4 Comparability: 2 Outcome: 2
Fasano et al. [14], 2013	Italy	cohort study	LBT and GBT	cases: 18/33(54.5%) controls: 6/30 (20%)	not stated	cases: 67.8 ± 8.5 controls: 65.4 ± 4.8	cases: 18/15 SIBO + : 11/7 SIBO-: 7/8 controls:13/17	cases: 11.7 ± 5.0 controls: none	Selection: 3 Comparability: 2 Outcome: 3
Dobbs et al. [15], 2013	UK	cross-sectional	LBT	40/66 (60.6%)	25 g lactulose load is orally administered, H2 increase > 20 ppm on 2 consecutive measurements within 2 h	Overall 63 (46, 82)	Overall 41/25	Overall 6.8 (4.5, 8.7)	Selection: 4 Comparability: 2 Outcome: 2
Dobbs et al. [16], 2012	UK	cross‐sectional	LBT	34/51 (66.7%)	25 g lactulose load is orally administered, H2 increase > 20 ppm on 2 consecutive measurements within 2 h	65 (48,81)	32/19	6.5 (4.4,8.9)	Selection: 4 Comparability: 2 Outcome: 2
Gabrielli et al. [17], 2011	Germany	cohort study	GBT	cases: 26/48(54.2%) controls:3/36 (8.3%)	50 g glucose load is orally administered, H2 increase > 12 ppm above basal value within 2 h	cases: 66 ± 10 SIBO + : 67 ± 10 SIBO-: 63 ± 9 controls: 64 ± 9	cases: 23/25 SIBO + : 14/12 SIBO-: 9/13 controls:16/20	SIBO + : 10.6 ± 3.6 SIBO-: 7.4 ± 2.7 controls: none	Selection: 4 Comparability: 2 Outcome: 2
Charlett et al. [18], 2009	UK	cross‐sectional	LBT	24/40(60%)	25 g lactulose load is orally administered, H2 increase > 20 ppm on 2 consecutive measurements within 2 h	60.8 (41.0–80.5)	not stated	not stated	Selection: 3 Comparability: 2 Outcome: 2
Davies et al. [19], 1996	UK	cohort study	LBT	cases:10/15(66.7%) controls:3/15(20%)	10 g lactulose load is orally administered, a rise of at least 5 ppm above fasting levels in the end expiratory hydrogen concentration	cases: 73.9 (68.3–80.5) controls: 73.6 (67.6–80.3)	cases: 6/9 controls: 6/9	not stated	Selection: 3 Comparability: 2 Outcome: 1

*LHB* lactulose breath test, *GBT* glucose breath test, *ppm* parts per million, *SIBO* small intestinal bacterial overgrowth

### Prevalence of SIBO in patients with PD

**Fig. 2:**
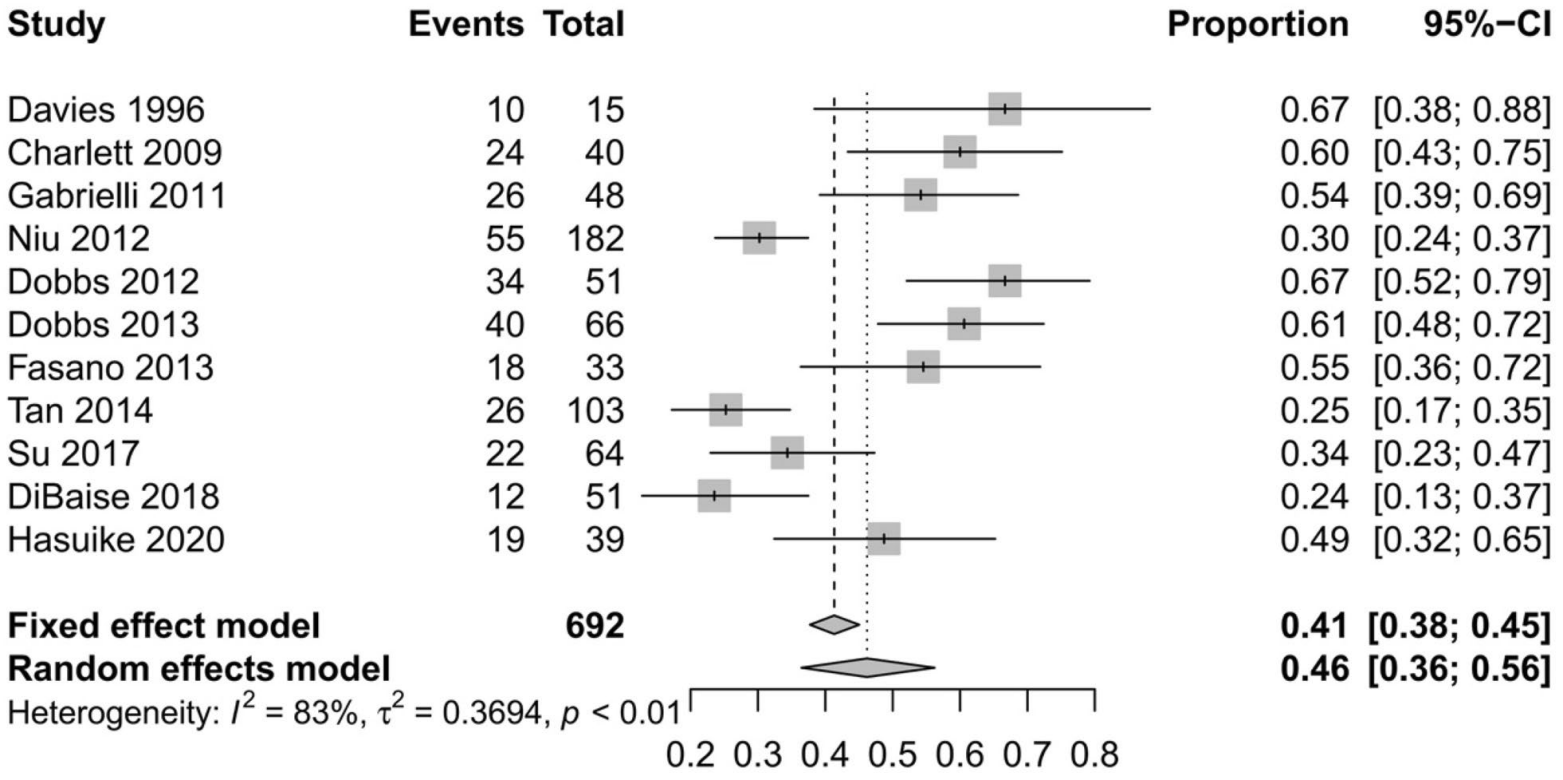
Forest plot of the pooled prevalence of SIBO in PD

**Fig. 3:**
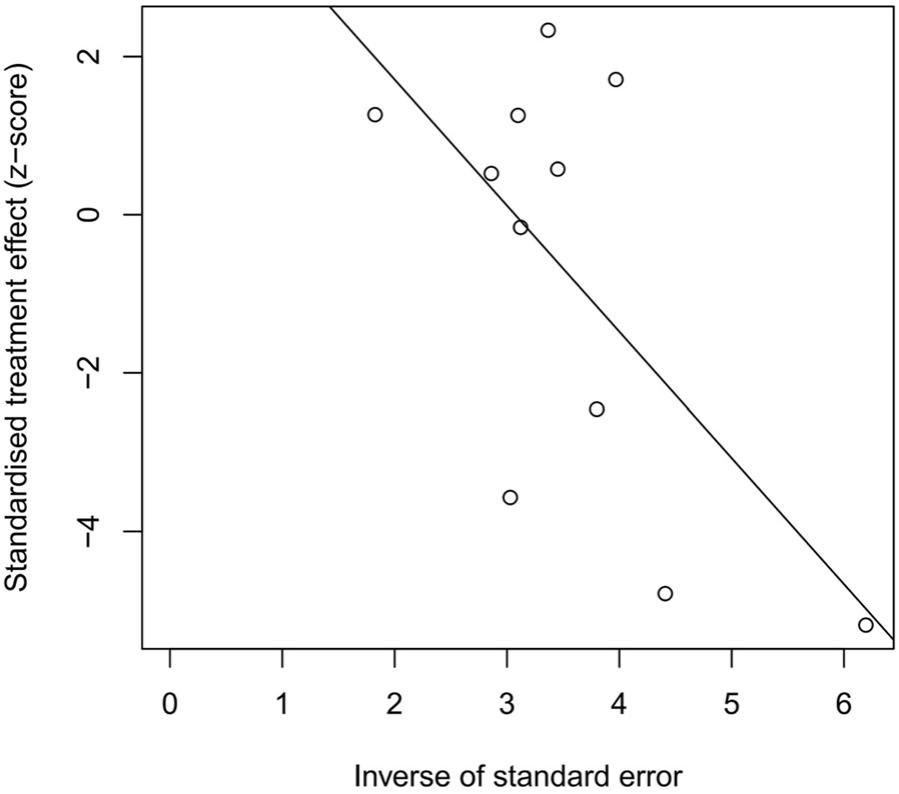
Egger’s test showing the publication bias of the pooled prevalence of SIBO in PD (p = 0.0657)

**Fig. 4:**
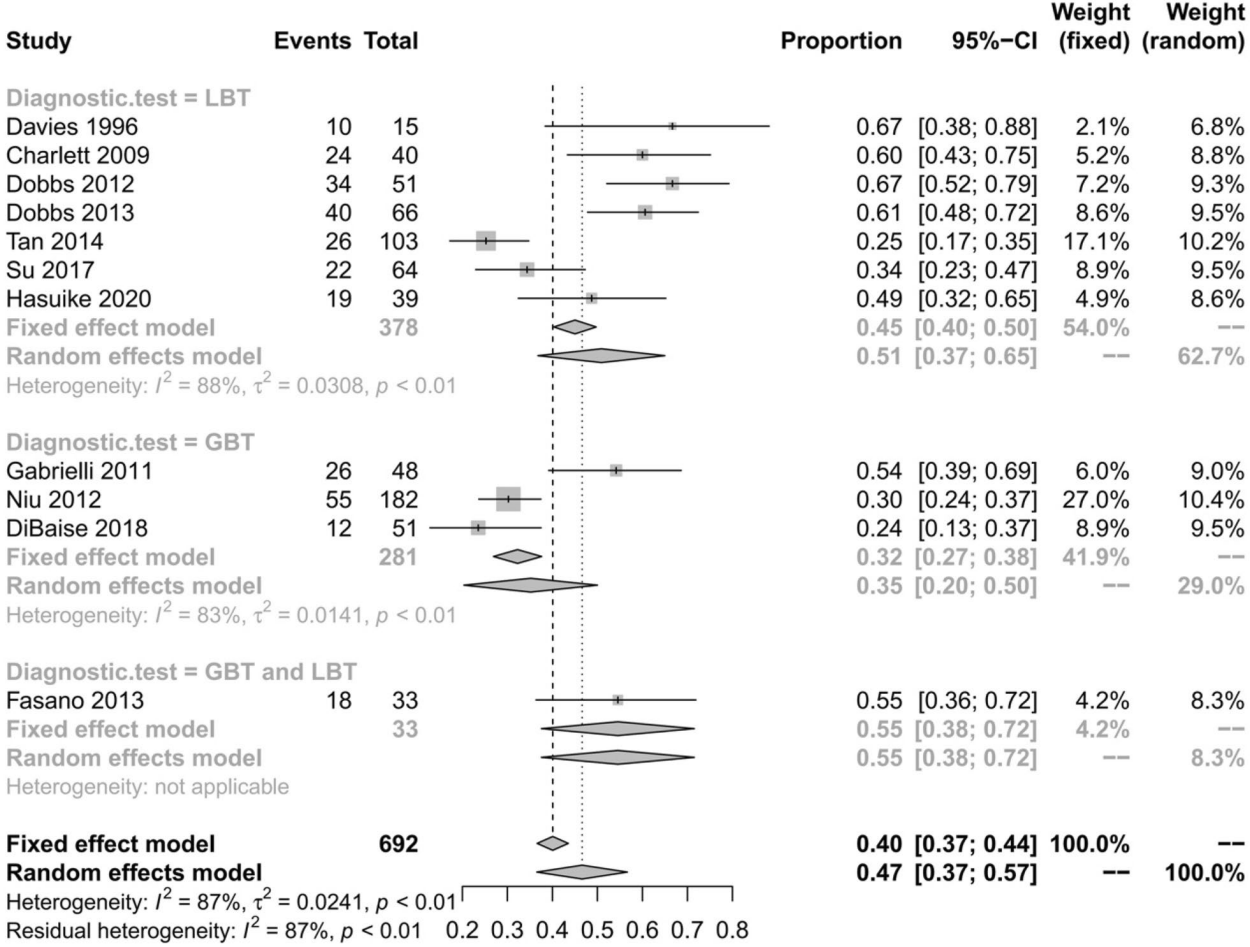
Forest plot of the prevalence of SIBO in PD based on the SIBO diagnostic test

**Fig. 5:**
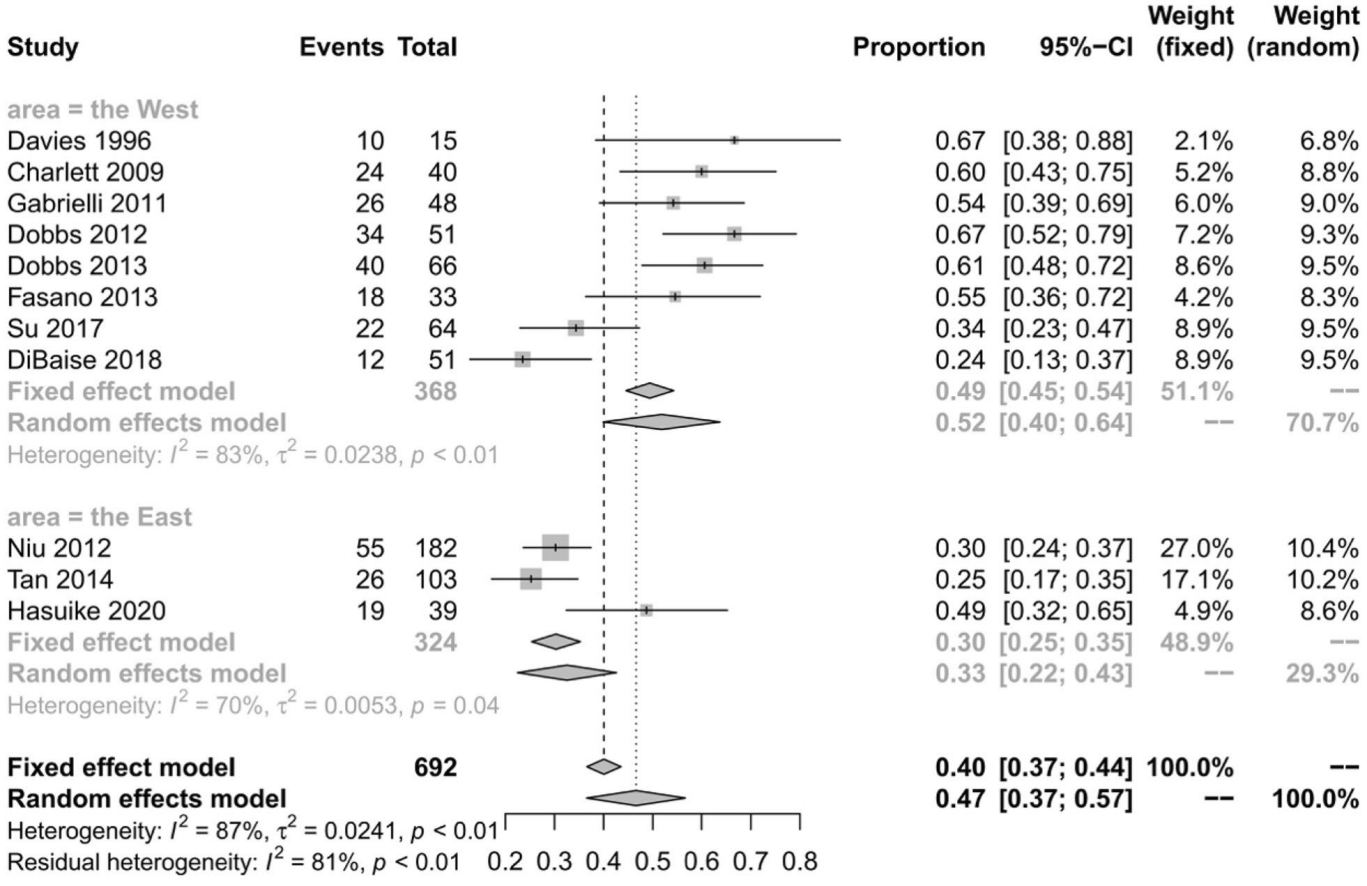
Forest plot of the prevalence of SIBO in PD based on geographic areas

All eleven studies [7, 10–19] reported the prevalence of SIBO in patients with PD. Overall, the pooled prevalence was 46% (95% CI 36–56) (Fig. 2). The highest prevalence of SIBO was 67% in PD patients diagnosed by LBT [16, 19], and the lowest prevalence was 24% in PD patients diagnosed by GBT [11]. A random-effects model was applied given the heterogeneity (I^2^ = 83%) detected among the studies. The results of Egger’s test indicated no publication bias (p = 0.0657) (Fig. 3). To explore the variability in the prevalence among the studies, we performed a subgroup analysis based on the type of SIBO diagnostic test. The subgroup analysis showed that the prevalence of SIBO was greater in studies in which patients were diagnosed by the LBT (51%, 95% CI 37–65) than in those in which patients were diagnosed by the GBT (35%, 95% CI 20–50), and the prevalence of SIBO in PD patients was highest (55%, 95% CI 38–72) in studies in which SIBO was diagnosed by the LBT and GBT (Fig. 4). Finally, in subgroup analysis based on geographic area, the prevalence of SIBO was 52% (95% CI 40–64) in patients from Western countries and 33% (95% CI 22–43) in patients from Eastern countries (Fig. 5).

### Prevalence of SIBO in PD patients compared with controls

**Fig. 6:**
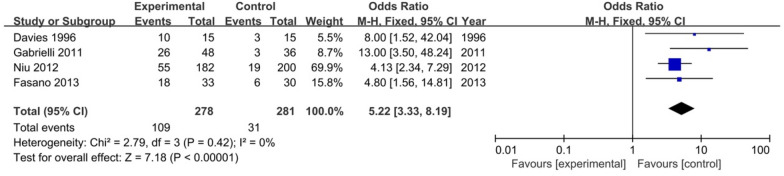
Forest plot of odds ratios of SIBO in PD patients compared with controls

Four case–control studies [12, 14, 17, 19] reported the prevalence of SIBO in 278 Parkinson's disease patients compared with 281 healthy controls. The pooled OR of SIBO in PD patients compared with healthy controls was 5.22 (95% CI 3.33–8.19, p < 0.00001) (Fig. 6). We used the fixed-effects model, and no heterogeneity was noted between the studies (I^2^ = 0, p = 0.42). However, we could not perform Egger’s test because relatively few studies were included.

### Predictors of SIBO in patients with PD

In our study, there were no obvious predictors for SIBO in PD patients. Five studies [10, 12–14, 17] including 401 PD patients assessed the link between bloating and SIBO. The prevalence of bloating in PD patients was not significantly different from that in patients without SIBO with a pooled OR of 1.67 (95% CI 0.65–4.27, p = 0.28). Three studies [10, 13, 17] examined disease duration as a predictor of SIBO in PD; the pooled OR of disease duration among the PD individuals with SIBO compared to those without SIBO was − 0.25, and the difference did not reach statistical significance (95% CI − 3.64 to 3.14; p = 0.88). Similarly, no significant differences in the prevalence of constipation [10, 12, 13, 17] and diarrhoea [10, 12, 13, 17] were noted between PD patients with and without SIBO with ORs of 0.38 (95% CI 0.08–1.78, p = 0.22) and 1.06 (95% CI 0.58–1.91, p = 0.86), respectively (Table 2).

**Table 2:** Results of the meta-analyses in predictors

Predictors	No. of studies (study size)	P-value	I^2^	OR (95% CI)
Bloating	5(401)	0.28	72%	1.67 [0.65–4.27]
Duration	3(186)	0.88	91%	− 0.25 [− 3.64–3.14]
Constipation	4(368)	0.22	85%	0.38 [0.08–1.78]
Diarrhea	4(368)	0.86	0	1.06 [0.58–1.91]

## Discussion

The causes of SIBO include a breakdown of the antibiotic barrier, abnormal gastrointestinal motility, and intestinal anatomical abnormalities. SIBO can trigger an inflammatory response in the intestinal mucosa and increase intestinal permeability. Increased intestinal permeability leads to mucosal immune system exposure to bacterial products, such as endotoxins, thereby increasing the expression of alpha-synuclein [20]. Alpha-synuclein can destroy the integrity of the blood–brain barrier and promote neuroinflammation and injury in the substantia nigra pars compacta [21]. Intestinal bacteria also enhance the inflammatory effect of alpha-synuclein by initiating a natural immune response, causing the misfolding of alpha-synuclein, which results in neurotoxic effects and dopamine neuron apoptosis. These events ultimately lead to the occurrence of PD [22]. PD can affect the autonomic nervous system, and autonomic nervous system dysfunction can lead to gastrointestinal dysfunction. Gastrointestinal symptoms common and precede motor symptoms. In addition, many drugs used to treat dyskinesia can cause gastrointestinal dysfunction [23], further leading to SIBO. SIBO can cause fluctuations in the subsequent absorption of those drugs, which affects the treatment of PD [24, 25]. According to the above mechanisms, studies have shown that faecal microbiota transplantation and probiotics may represent adjuvant therapies for PD [26, 27].

This study is the first systematic review and meta-analysis to summarize the latest evidence of the association between SIBO and PD. The pooled prevalence of SIBO was 46% in PD patients. The prevalence of SIBO appears to be related to the type of diagnostic test used with a greater prevalence associated with the LBT (51%) than the GBT (35%). Regional differences are also noted. The prevalence of SIBO in Western countries (52%) is greater than that in Eastern countries (33%). These results suggest a strong correlation between SIBO and PD. We also examined the relationship between diarrhoea, bloating, and constipation and the occurrence of SIBO in PD patients. Unfortunately, none of these indicators was statistically significant. Thus, no valid predictors have been identified to date.

The prevalence of SIBO varies with different diagnostic methods. In our study, the prevalence of SIBO diagnosed by the LBT was greater than that diagnosed by the GBT. The difference in the prevalence according to the different diagnostic methods may be due to the rapid transport of lactulose in the intestinal tract, which reached the colon quickly, resulting in excessive hydrogen. This may lead to false-positive results [28]. In addition, our study found that the prevalence of SIBO in PD patients was greater in Western countries compared with Asian countries. One possible explanation is that different countries have different eating habits. Fatty and high-carbohydrate foods in Western diets can reduce the abundance of intestinal microbes and increase the numbers of anaerobic bacteria and enteric bacilli. Additionally, differences in metabolism and systemic immune function are noted between people in different regions [5].

Malnutrition and osteoporosis have been reported to be characteristics of PD patients with SIBO in previous studies. When bacteria in the small intestines overgrow, unconjugated bile acids become dominant, bile acid synthesis is inhibited, and the bile acid level is reduced. Decreased lipid absorption occurs when bile acid levels are decreased, and the low triglycerides (TG) levels can be explained by this phenomenon [10]. Tan et al. [29] reported that PD patients exhibit reduce body fat with relatively preserved skeletal muscle mass. Such poor lipid absorption due to SIBO may explain the relationship between PD and weight loss. In addition, PD is independently associated with lower bone mineral density (BMD) [30, 31]. Reductions in bile acid function due to SIBO also will impair the absorption of lipid-soluble vitamins, mainly vitamin D. This feature may be related to osteoporosis and fractures in PD patients.

There are some limitations in our meta-analysis. The sample size was relatively small. Heterogeneity may have existed given the use of different diagnostic methods and populations from different locations, which affects the reliability of the results.

## Conclusion

Our meta-analysis identified a strong association between SIBO and PD with approximately half of PD patients testing positive for SIBO. These relationships were significantly different according to type of diagnostic test and geographic area. Therefore, we must pay close attention to enteric microorganisms to prevent nervous system diseases.

## Data Availability

The data and material are available from the corresponding author upon request.
